# Biologic Therapy in Severe Asthma: A Phenotype-Driven and Targeted Approach

**DOI:** 10.3390/jcm14134749

**Published:** 2025-07-04

**Authors:** Maria D’Amato, Daniela Pastore, Chiara Lupia, Claudio Candia, Andrea Bruni, Eugenio Garofalo, Federico Longhini, Angelantonio Maglio, Albino Petrone, Alessandro Vatrella, Girolamo Pelaia, Corrado Pelaia

**Affiliations:** 1 Respiratory Department, Monaldi Hospital AO Dei Colli, Federico II University, 80131 Naples, Italy; marielladam@hotmail.it; 2 Department of Health Sciences, University “Magna Græcia” of Catanzaro, 88100 Catanzaro, Italy; danielapastore11@gmail.com (D.P.); chiaralupia1996@gmail.com (C.L.); pelaia@unicz.it (G.P.); 3 Department of Biomedicine, Neurosciences and Advanced Diagnostics, University of Palermo, 90133 Palermo, Italy; claudio.candia@unina.it; 4 Department of Experimental and Clinical Medicine, University “Magna Græcia” of Catanzaro, 88100 Catanzaro, Italy; andreabruni@unicz.it; 5 Department of Medical and Surgical Sciences, University “Magna Græcia” of Catanzaro, 88100 Catanzaro, Italy; eugenio.garofalo@unicz.it (E.G.); flonghini@unicz.it (F.L.); 6 Department of Medicine, Surgery and Dentistry, University of Salerno, 84084 Salerno, Italy; amaglio@unisa.it (A.M.); avatrella@unisa.it (A.V.); 7 Department of Respiratory Diseases, Annunziata Hospital, 87100 Cosenza, Italy; alb.petronedoc@gmail.com

**Keywords:** severe asthma, biologic therapy, eosinophils, FeNO, IgE, personalized medicine

## Abstract

Asthma is a highly heterogeneous respiratory disease that, in its severe forms, is characterized by persistent symptoms, frequent exacerbations, and a significant impact on patients’ quality of life. Despite high-dose inhaled corticosteroids and long-acting bronchodilators, a subset of patients remains uncontrolled, necessitating advanced therapeutic strategies. The advent of biologic therapies has revolutionized the management of severe asthma, offering targeted interventions based on the underlying inflammatory endotypes, primarily T2-high and T2-low. However, selecting the most appropriate biologic remains challenging due to overlapping phenotypic features and the limited availability of validated biomarkers. This narrative review explores the clinical utility of key biomarkers, including blood eosinophils, fractional exhaled nitric oxide (FeNO), periostin, and total and specific IgE, in guiding biologic therapy. All the information provided is based on an extensive literature search conducted on PubMed. We also examine the clinical characteristics and comorbidities that influence therapeutic choices. Furthermore, we present a practical decision-making platform, including a clinical table matching phenotypes with biologic agents, such as omalizumab, mepolizumab, benralizumab, dupilumab, and tezepelumab. By integrating biomarker analysis with clinical assessment, based on current guidelines and our extensive real-life experience, we aim to offer a logical framework to help clinicians select the most suitable biologic treatment for patients with uncontrolled severe asthma. Future research should focus on identifying novel biomarkers, refining patient stratification, and evaluating long-term outcomes to further advance precision medicine in the management of severe asthma.

## 1. Introduction

Asthma is a heterogeneous, chronic, inflammatory respiratory disease characterized by airway inflammation and respiratory symptoms, including wheezing, difficult breathing, chest tightness, and coughing, associated with airflow limitation [1]. About 5–10% of asthmatic patients remain uncontrolled despite common treatments [2]. These patients experience frequent exacerbations and often require oral corticosteroids, which significantly decrease their quality of life. Various phenotypes have been identified so far: allergic eosinophilic, non-allergic eosinophilic, mixed eosinophilic and neutrophilic, neutrophilic, and paucigranulocytic [3]. Pathways driven by T2-high inflammation can benefit from numerous biological therapies. Many cytokines serve as targets for currently available biological treatments. When exposed to allergens, pollutants, viral agents, and bacteria, airway epithelial cells become more fragile and permeable to environmental agents that can cause damage. Consequently, epithelial cells release innate cytokines, known as alarmins, including thymic stromal lymphopoietin (TSLP), interleukin-25 (IL-25), and interleukin-33 (IL-33). Such alarmins promote the differentiation of T helper 2 cells and the activation of type 2 innate lymphoid cells (ILC2). These two cell types produce numerous type-2 cytokines, including interleukin-5 (IL-5), which is associated with the maturation and differentiation of eosinophils; interleukin-4 (IL-4), which drives IgE production from B cells; and interleukin-13 (IL-13), which induces the expression of the inducible nitric oxide synthase in airway epithelial cells, resulting in an increased production of fractional exhaled nitric oxide (FeNO). IL-13 is also involved in mucus hypersecretion and airway hyperresponsiveness [4,5]. On the other hand, low-profile T2 asthma involves the activation of T helper 1 (Th1) and T helper 17 (Th17) cells, leading to increased levels of interleukin-17A (IL-17A) [6]. Typically, T2-low asthma occurs in adulthood and is often associated with several comorbidities such as obesity and gastroesophageal reflux. The primary purpose of this review is to summarize currently available data on each biological drug, beginning with an analysis of the key biomarkers which are used to choose the most effective treatment for each asthmatic patient (Table 1). Although biological agents have been developed to target specific asthma endotypes, it is often challenging for physicians to identify which patients are the best candidates for each therapy. This is especially true for patients whose clinical characteristics overlap among different endotypes, allowing for the selection of more than one biologic agent. Regardless of how carefully made, a physician’s first therapeutic decision may often lead to a suboptimal clinical response and drug discontinuation, indicating the need to switch to a different biologic. Although biologics are extremely precise, only a few biomarkers are available to help the choice of the best drug for our patients (Table 2). This review aims to assist clinicians in selecting a biological drug by evaluating biomarkers and integrating the patient’s clinical characteristics and prevalent symptoms. In particular, based on current guidelines and our long-term, real-life experience, we aim to provide a rational framework to guide clinicians towards the best possible option for choosing an appropriate biologic treatment for each patient with uncontrolled severe asthma. All information reported was obtained through an extensive literature search on PubMed, selecting the most relevant papers published between 2000 and 2025. We used the keywords “severe asthma”, “biomarkers”, “comorbidities”, and “biologic drugs” for our search.

## 2. Biomarkers

### 2.1. Fractional Exhaled Nitric Oxide

Quantification of fractional exhaled nitric oxide (FeNO) is valuable in managing severe asthma by providing insights into airway inflammation and guiding treatment decisions [7,8]. It is a non-invasive parameter of bronchial T2 phenotype and epithelial damage. In 2011, the American Thoracic Society (ATS) published its guide on interpreting FeNO results [9]. It is suggested to define levels < 25 parts per billion (ppb) in adults (<20 ppb in children) as low FeNO and levels > 50 ppb (>35 ppb in children) as high [7]. Airway inflammation is often more pronounced and resistant to standard therapies in individuals with severe asthma, making accurate assessment crucial for effective management. FeNO measurement provides a noninvasive and quantitative method for assessing airway inflammation, particularly eosinophilic inflammation, which is a common feature of severe asthma [4,8].

High levels of FeNO are associated with ongoing eosinophilic inflammation in the airways, suggesting a potential need for targeted anti-inflammatory therapies, including biologics targeting eosinophils [10,11]. Its levels seemingly do not vary with anti-IL-5 (mepolizumab) or anti-IL-5R (benralizumab) therapy [12], although some variability has been reported, despite no apparent consequence on functional and asthma control parameters [13]. Interestingly enough, a recent work by Soendergaard et al. [14] highlighted that a FeNO response after only four months of treatment with anti-IL-5 agents could predict remission in patients with severe asthma. Dupilumab is a monoclonal antibody that binds to IL-4R, thereby neutralizing the biological actions of IL-4 and IL-13. It works well when the basal FeNO level is >20. The most significant response occurs for values > 50 ppb [15,16]. Another drug capable of reducing FeNO levels is tezepelumab [17].

### 2.2. Blood Eosinophil Count

One of the most essential biomarkers is the total eosinophil count in peripheral blood. It is easily obtainable and widely used. Elevated blood eosinophil counts are associated with increased airway inflammation and are often observed in individuals with allergic asthma or severe disease. They predict the risk of exacerbations in both children and adults, as well as the response to biological treatments [18,19,20]. Asthma is characterized by the recruitment of eosinophils into the large airway wall and lumen, resulting in mucus plugging and airway thickening. High blood eosinophil levels are linked to poorer asthma control, increased frequency of exacerbations, and decreased lung function [18,21]. Therefore, individuals with increased eosinophil levels may benefit from therapies that specifically target eosinophilic inflammation, including monoclonal antibodies against interleukin-5 (IL-5) or IL-5R [22]. An increase in peripheral eosinophils in the absence of symptoms does not indicate asthma. Still, a level generally higher than 300 cells/microliter of blood, in association with asthmatic symptoms, continuous or cyclic use of oral corticosteroid (OCS), or the presence of bronchial obstruction is indicative of severe eosinophilic bronchial asthma [21,23,24,25].

Together with FeNO, blood eosinophils represent the most useful biomarkers for detection of the overall risk of asthma attacks and exacerbations [26]. Therefore, downregulation of these biomarkers should be one of the most important goals of add-on biologic therapies of severe asthma.

### 2.3. Periostin

Periostin is a protein that plays a significant role in the pathogenesis of asthma, particularly in severe asthma and airway remodeling. It is primarily expressed in the extracellular matrix of various tissues, including the airways, where it regulates tissue repair, remodeling, and inflammation [2]. In individuals with asthma, periostin levels are often increased, especially in those with eosinophilic inflammation and severe disease. These changes can lead to airway narrowing, decreased lung function, and increased susceptibility to exacerbations [27,28]. Measuring periostin levels in the blood or sputum can serve as a biomarker for eosinophilic inflammation and airway remodeling in asthma. High periostin levels have been associated with more severe asthma phenotypes, an increased risk of exacerbations, and poorer responses to standard asthma therapies, highlighting its potential utility in guiding treatment decisions, particularly the use of biologic therapies addressing eosinophilic inflammation [22,29]. Biologic agents, including monoclonal antibodies targeting interleukin-13 (IL-13) such as dupilumab, have shown efficacy in reducing periostin levels and improving asthma control in patients with severe eosinophilic asthma. By targeting the pathways involved in periostin expression and airway remodeling, these biologic therapies offer a more focused approach to asthma management [29,30]. Anyway, because of high variable levels and lack of standardization, periostin is not currently believed to be a valuable biomarker of severe asthma.

### 2.4. Sputum Eosinophils

Sputum eosinophil counts are valuable biomarkers for assessing and managing asthma, similar to blood eosinophil counts [31]. By analyzing sputum samples, healthcare providers can directly evaluate airway inflammation and tailor treatment strategies to address the underlying inflammatory endotype of the disease [32]. The use of sputum is advantageous for patients with severe asthma undergoing chronic treatment with OCS when blood eosinophil levels are low or, in cases where an exacerbation occurs during biological therapy, to assess the potential presence of eosinophils or other cellular elements [25,31,32,33].

### 2.5. Immunoglobulins E

Serum levels of immunoglobulins E (IgE) are relevant biomarkers of T2 inflammation. IgE levels can serve as a biomarker for evaluating and managing severe asthma, particularly in identifying patients with allergic inflammation [32]. In patients with allergic asthma, characterized by airway inflammation driven by allergen-specific IgE antibodies, measuring IgE levels can help identify those with an allergic phenotype and guide the selection of appropriate therapies targeting allergic inflammation [22,29,32,34]. Additionally, elevated IgE levels may be related to specific asthma comorbidities, including allergic rhinitis, atopic dermatitis, or allergic bronchopulmonary aspergillosis (ABPA) [35,36]. Assessing IgE levels can help identify patients at increased risk for these comorbidities and drive the management of underlying allergic conditions that may contribute to asthma severity [31,35]. Although serum IgE are very useful to identify the allergic phenotype, however they do not correlate with the degree of asthma severity. Moreover, IgE changes induced by biologic treatments do not always reflect the extent of clinical and functional improvements.

### 2.6. Emerging Potential Biomarkers

In addition to the above-mentioned, widely used biomarkers, other systemic indicators of type-2 inflammation could include serum levels of IL-5, IL-13, and thymus and activation-regulated chemokine (TARC) [37]. Indeed, within the network of pro-inflammatory pathways underlying T2-high asthma, these biomarkers are located downstream of the involvement of alarmins, which are the upstream inducers of innate and adaptive immune responses [37]. Further advances towards a better characterization of asthmatic phenotypes/endotypes, aimed to provide a stronger platform for precision medicine, could be offered by multi-omic approaches such as genomics, epigenomics, transcriptomics, proteomics, metabolomics, and breathomics (exhaled condensate) [38].

Sensitivity, specificity, and real-world clinical utility of currently used biomarkers have been summarized in Table 1.

## 3. Clinical Characteristics

Severe asthmatic subjects can be clinically differentiated on the basis of their features and specific comorbidities, which often accompany a particular phenotype. To characterize the heterogeneous population of patients with severe asthma, as well as to facilitate the best choice of a personalized biological therapy, we propose a subdivision into the following groups: (i) atopic asthmatics with rhinitis or rhinosinusitis; (ii) asthmatic patients with nasal polyposis; (iii) patients with recurrent exacerbations requiring a frequent use of OCS; (iv) patients with fixed bronchial obstruction; (v) patients with severe asthma and obesity; (vi) patients with low biomarker levels. The detailed analysis of the clinical features of severe asthma must necessarily be integrated by their correlations with biomarker levels. For example, serum IgE levels should be considered in association with the clinical importance of allergic sensitization. Moreover, FeNO levels and blood eosinophils counts should be measured and monitored with regard to the risk of asthma attacks and exacerbations.

### 3.1. Allergic Asthma with Rhinitis or Rhinosinusitis

A combination of upper and lower airway symptoms, allergic sensitization and comorbidities characterizes severe asthma in atopic patients with rhinitis or rhinosinusitis. The atopic status can be assessed based on serum total and specific IgE levels, identified in response to a panel of common aeroallergens, or through the positive result of a skin prick test (SPT) to such aeroallergens [39]. Airway inflammation triggered by aeroallergens characterizes allergic asthma. T-helper cell type 2 (Th2) cytokines, including interleukin (IL)-4, IL-5, and IL-13, drive this inflammatory pattern. These cytokines trigger cascades of downstream events, including airway inflammation, airway eosinophilia, and increased immunoglobulin E (IgE) synthesis [31,40]. Allergic rhinitis (AR) is an immunoglobulin E (IgE)-mediated inflammation of the nasal mucosa. AR affects an estimated 400 million individuals worldwide, making it the most prevalent atopic disease, with its prevalence increasing globally [41,42]. Furthermore, patients with atopic asthma and rhinitis may be more likely to develop other allergic conditions, including atopic dermatitis (eczema) or allergic conjunctivitis (eye allergies). These comorbidities complicate disease management and have a significant impact on overall health and well-being [41,42].

Optimal management often requires a comprehensive approach that targets both upper and lower airway inflammation. Chronic rhinosinusitis (CRS) is an inflammation of the nose and paranasal sinuses lasting at least 12 weeks. It is characterized by anterior or posterior rhinorrhea, nasal obstruction, facial pain and/or pressure, and alterations in the sense of smell [43,44]. Several cross-sectional studies have shown that rhinitis and asthma often co-occur. It is challenging to determine whether rhinitis is the initial manifestation of respiratory allergy in a patient who may eventually develop asthma, or whether nasal disease is directly contributing to the development of asthma. Long-term epidemiologic studies of patients with allergic rhinitis, including assessments of bronchial responsiveness, will be critical in answering this question [45,46,47,48].

Another comorbidity which can coexist with severe asthma is food allergy, caused by common atopic mechanisms [49]. Therefore, the eventual occurrence of food allergy should be carefully investigated in patients with severe asthma. Because of the pivotal pathogenic role played by IgE in such conditions, these immunoglobulins represent key biomarkers and strategic molecular targets for biologic therapies [50].

### 3.2. Asthmatic Patients with Nasal Polyposis

Asthmatic patients with nasal polyposis represent a distinct subgroup within the broader asthma population. When nasal polyposis coexists with asthma, it can influence the clinical presentation and management of both conditions [51]. Asthmatic patients with nasal polyposis often exhibit persistent airway inflammation, leading to frequent exacerbations, reduced lung function, and poor asthma control [51]. A subset of asthmatic patients with nasal polyposis may also complain of aspirin-exacerbated respiratory disease (AERD), also known as aspirin sensitivity. These patients experience worsening of asthma symptoms, nasal congestion, and respiratory reactions (including bronchoconstriction) upon ingestion of aspirin or other nonsteroidal anti-inflammatory drugs (NSAIDs) [52]. The coexistence of asthma and nasal polyposis may exacerbate such clinical manifestations, thus leading to a more substantial global disease burden [51,52]. Currently, the severe asthma and nasal polyposis phenotype is the typical expression of eosinophilic inflammation, which implies the use of anti-IL-5 or anti-IL-5 receptor biologic therapies, as well as anti-IL-4R receptor therapy. Some biological drugs have therapeutic indications for both severe asthma and nasal polyposis, including dupilumab, omalizumab and mepolizumab [53]. Therefore, the choice of the most appropriate of such biologics for each patient may be helped by the prevalent biomarker (namely, IgE for omalizumab, blood eosinophils for mepolizumab, and FeNO for dupilumab).

### 3.3. Patients with Recurrent Exacerbations and Frequent Use of OCS

Subjects with frequent exacerbations and regular use of OCS represent a high-risk subgroup of asthmatic patients who require specialized care and multidisciplinary management [54]. Their asthma may be challenging to be controlled, resulting in ongoing airway inflammation and heightened disease activity [55,56]. Persistent airway inflammation is a hallmark of asthma in these patients, usually associated with increased levels of eosinophils or other markers of inflammation in blood, sputum, or exhaled breath [19,21,33]. Persistent airway inflammation and recurrent exacerbations contribute to irreversible airway remodeling, leading to decreased lung function and impaired respiratory health [56]. Often these patients also suffer from pathologies related to the frequent use of OCS: corticosteroid dependency and associated adverse effects including weight gain, osteoporosis, diabetes, hypertension, and immunosuppression. Steroid-induced comorbidities further complicate disease management and reduce the overall quality of life [56]. Thus, the selection of the best option for biologic treatment should take into account the effectiveness of each monoclonal antibody in decreasing OCS intake.

### 3.4. Patient with Fixed Bronchial Obstruction

Fixed bronchial obstruction is characterized by a reduced airflow that persists even after maximal bronchodilation. Chronic inflammation induces remodeling, including thickening of the basement membrane and other changes that cause non-reversible airway obstruction [57,58]. Fixed bronchial obstruction can lead to complications including recurrent respiratory infections, exacerbations of underlying lung disease, worsening dyspnea, impaired exercise tolerance, and reduced global lung function. These complications may further exacerbate symptoms and decrease the quality of life [58]. Common biologics include monoclonal antibodies targeting IL-5 (e.g., mepolizumab, reslizumab) or its receptor (benralizumab), IL-4/IL-13 receptors (dupilumab), and immunoglobulins E (omalizumab); however, their effectiveness in patients with fixed bronchial obstruction may be limited [58,59,60]. This limitation is due to fixed obstruction being often associated with structural changes in the airways, including fibrosis and airway remodeling, which cannot be fully reversed by targeted anti-inflammatory therapies. Patients with fixed bronchial obstruction may exhibit an eosinophilic phenotype characterized by increased blood or sputum eosinophils. In such patients, biological therapies targeting eosinophils, including mepolizumab, reslizumab and benralizumab, may still be considered, as they can help reduce eosinophilic inflammation and improve asthma control, even in the presence of fixed airflow limitation [61,62]. Very recently, dupilumab has been demonstrated to enhance airway volume and flow, corresponding to improved lung function and asthma control [63].

### 3.5. Severe Asthma and Obesity

Severe asthma in obese individuals may be characterized by persistent airway inflammation, airflow limitation, and reduced lung function [64]. Adipose tissue is metabolically active and produces pro-inflammatory cytokines, including tumor necrosis factor-alpha (TNF-α), IL-6, and leptin, which may contribute to both systemic and airway inflammation in obese individuals with asthma. This chronic low-grade inflammation may exacerbate asthma symptoms and contribute to disease severity [65]. Managing severe asthma in obese individuals requires a comprehensive approach that addresses both asthma and obesity-related comorbidities. Lifestyle interventions aimed at weight loss, along with optimized asthma management, are essential for improving outcomes in this vulnerable population [65]. Clinical trials evaluating the efficacy and safety of tezepelumab in severe asthma have enrolled patients with various demographic characteristics, also including obese individuals. Analyses of trial data may provide insights into the effectiveness of tezepelumab in obese patients with severe asthma. Tezepelumab’s broad anti-inflammatory effects, due to TSLP targeting, may offer potential benefits in obese individuals with severe asthma by addressing underlying inflammation and improving asthma control, even in the absence of specific biomarkers [66,67].

### 3.6. Severe Asthma Associated with Low Biomarker Levels

Some patients with severe asthma may not have readily identifiable biomarkers, making it difficult to determine the underlying inflammatory mechanisms driving their disease. Research into novel therapies for severe asthma, including biologic agents targeting alternative inflammatory pathways, is ongoing. These therapies may offer new treatment options for patients with severe asthma in the absence of traditional biomarkers [66]. Tezepelumab is a monoclonal antibody targeting thymic stromal lymphopoietin (TSLP), a key innate cytokine involved in the initiation and maintenance of allergic and non-allergic airway inflammation. Several randomized clinical trials have shown that tezepelumab can exert positive therapeutic effects in the treatment of severe asthma, particularly in patients with uncontrolled disease despite high-dose inhaled corticosteroids (ICS) and other controller medications, even in the absence of specific biomarkers [17,66,67,68]. In addition to their role in the onset and maintenance of type 2 asthma, alarmins seem to play also a relevant part in T2-low neutrophilic bronchial inflammation, primarily driven by pro-inflammatory pathways activated by Th17 lymphocytes. Notably, alarmins are capable of stimulating dendritic cells to release interleukin-6 (IL-6) and interleukin-23 (IL-23), which are essential for Th17 skewing. Therefore, alarmins are currently believed to be suitable targets for biologic therapies of T2-low asthma [69].

## 4. Current Biological Treatments

### 4.1. Omalizumab

Omalizumab was the first biological drug used for severe asthma. It was approved by the Food and Drug Administration (FDA) in 2003, and subsequently by the European Medicines Agency (EMA) in 2005 [70]. It is a humanized monoclonal antibody that binds to the constant portion (Fc) of IgE, thus inhibiting the binding of this class of immunoglobulins to both high- and low-affinity IgE receptors present on mast cells, basophils, eosinophils, dendritic cells, and airway structural cells. Omalizumab has been approved for adults and children over 6 years old with severe allergic asthma. This biologic has also been licensed for chronic idiopathic urticaria and chronic rhinosinusitis with nasal polyposis (CRSwNP) [71]. Omalizumab is administered subcutaneously every 2 to 4 weeks, at a dose dependent on the patient’s weight and serum IgE levels. Although serum IgE levels are necessary to determine drug dosage, they do not correlate with a greater clinical response to therapy. Instead, numerous data from clinical trials highlight that levels of other biomarkers, such as FeNO and blood eosinophil counts (>250 cells/µL), are more strongly associated with clinical response, primarily consisting of decreased exacerbations. Nevertheless, it is essential to note that real-world studies have not confirmed these findings [72,73]. This biological treatment has been shown to reduce asthma exacerbations and the use of oral corticosteroids. It can also increase FEV_1_ and, as demonstrated by a few real-world studies using AQLQ (Asthma Quality of Life Questionnaire), may enhance the quality of life in some asthmatic patients [74,75].

### 4.2. Mepolizumab

Mepolizumab was the first humanized monoclonal antibody authorized for severe eosinophilic asthma. It targets interleukin 5 (IL-5), which is responsible for the proliferation, differentiation, and survival of eosinophils. Several studies have shown that mepolizumab reduces both blood and airway eosinophils [76]. This add-on treatment was approved for patients over 6 years in Europe and over 12 years in USA. Patients who may benefit from this therapy are those with blood eosinophil counts exceeding 150 cells/µL before the first administration and 300 cells/µL in the previous year, as well as those experiencing two asthma exacerbations in the past year requiring oral corticosteroids [77,78]. Many trials, including MENSA and SIRIUS, have demonstrated a reduction in the rate of exacerbations and effectiveness in decreasing OCS dosage in patients who rely on daily corticosteroids to maintain asthma control [79,80]. Besides treating severe eosinophilic asthma, mepolizumab is also authorized for eosinophilic granulomatosis with polyangiitis (EGPA) [81]. Additionally, both FDA and EMA have recently approved this biological drug for treating chronic rhinosinusitis with nasal polyps (CRwNP). Indeed, many patients with severe bilateral nasal polyps, unresponsive to medications and surgery, experienced a significant reduction in endoscopic nasal scores and nasal obstruction symptoms when treated with mepolizumab [82]. Real-world data confirm these results, demonstrating a significant decrease in symptom severity [83]. A recent multicenter study, named MESILICO, which involved eight pulmonology departments in Greece, showed that patients with late-onset severe asthma who exhibited eosinophilic and impaired reversibility phenotypes not only reported significant clinical improvements, but also underwent a decrease in airway remodeling indices after receiving mepolizumab treatment for one year [84].

### 4.3. Reslizumab

Reslizumab is a recombinant humanized IgG4 antibody that binds to IL-5, resulting in a reduction of blood and airway eosinophils. It was authorized for patients over 18 years with severe eosinophilic asthma, who remain uncontrolled despite high doses of ICS inhalers plus another inhaled medication. Reslizumab is recommended for individuals who experienced asthma flare-ups during the last 12 months and have ≥400 eosinophils/μL [85]. Some studies have shown a reduction in asthma exacerbations, along with improvements in lung function, in patients with high levels of blood eosinophils [85]. However, the efficacy of this drug in patients using OCS has not yet been assessed. Reslizumab is administered intravenously at a weight-based dose of 3 mg/kg every 28 days. Recently, an unsuccessful study based on subcutaneous formulation was conducted [86]. Reslizumab has also been shown to be effective in EGPA; however, this effect was only observed in a small cohort of patients in open-label and observational studies [87].

### 4.4. Benralizumab

Benralizumab is a humanized monoclonal antibody authorized in 2017, directed against the IL-5 receptor α subunit (IL-5Rα). As a competitive inhibitor of IL-5, benralizumab binds more strongly to the α subunit of IL-5R, which is expressed by mature basophils and eosinophil precursors. Benralizumab also acts through further mechanisms, inducing antibody-dependent cell-mediated cytotoxicity (ADCC) that results in the apoptosis of eosinophils, which is triggered by activated natural killer cells. Additionally, benralizumab promotes antibody-dependent cellular phagocytosis (ADCP) by macrophages, which eliminate eosinophils through phagocytosis and efferocytosis. Macrophage efferocytosis clears apoptotic eosinophils. Stimulatory cytokines like interferon-γ (IFN-γ) are released by benralizumab-activated NK cells to cause macrophage cytotoxicity via TNF-α, which may initiate TNF receptor 1 (TNFR 1)-dependent eosinophil apoptosis [88,89]. Patients with uncontrolled severe eosinophilic asthma and a blood eosinophil count of at least 300 cells/µL are eligible to use this biologic medication as add-on therapy [90,91]. For the first three times, a subcutaneous dose of 30 mg benralizumab is administered every 28 days; thereafter, benralizumab is injected every 56 days. Benralizumab has been shown in large trials to improve lung function, decrease the frequency of exacerbations, and reduce the need for OCS in patients with moderate to severe asthma [92]. By comparing benralizumab and placebo, both SIROCCO and CALIMA trials demonstrated that the incidence of exacerbations decreased by 17–40% in benralizumab-treated individuals with blood eosinophil counts < 300 cells/μL, while it decreased by 28–51% in those with blood eosinophil numbers ≥ 300 cells/μL [92,93]. Patients with OCS dependence were included in the ZONDA study, which showed a 50% decrease in OCS dose when benralizumab was compared to placebo [94]. The most accurate indicators of benralizumab response, according to a combined analysis of these studies, include adult-onset asthma, more than three exacerbations in the preceding year, nasal polyposis, and pre-bronchodilator FVC < 65% of expected [92]. The clinical benefits of benralizumab in lowering AER and OCS requirements, as well as in reducing symptom ratings and enhancing quality of life indicators, have now been confirmed in real-world cohorts [95].

### 4.5. Dupilumab

Dupilumab is a fully human monoclonal antibody authorized in 2018 that inhibits the IL-4 and IL-13 pathways by binding to the IL-4 receptor α-subunit, thereby exerting a mutual receptor antagonism of both IL-4 and IL-13. Dupilumab has been approved as an add-on maintenance treatment for asthmatic patients classified across GINA steps 4/5 and who present type 2 inflammation, defined by increased FeNO and/or blood eosinophils. The initial dose of dupilumab is administered subcutaneously as either two injections of 200 mg each (400 mg total), followed by one injection of 200 mg every 14 days, or as a starting dose of 600 mg (two injections of 300 mg each), followed by 300 mg every 14 days. The latter treatment plan is recommended for patients with atopic dermatitis or asthma who are entirely reliant on OCS [96]. After initially receiving approval to treat atopic dermatitis, dupilumab has shown efficacy in moderate to severe asthma [97,98]. Additionally, dupilumab is recommended for the treatment of nasal polyposis. Regardless of peripheral blood eosinophil count, some trials have demonstrated that dupilumab reduces the frequency of asthma exacerbations, improves lung function measures and asthma control test scores, and decreases the need for OCS in severe asthmatics [99]. A transient increment of blood eosinophilia at the beginning of dupilumab therapy has been sometimes reported; however, this might be due to impeded migration into tissues rather than hyperproduction [100]. Furthermore, decreased levels of T2 inflammatory indicators including FeNO, serum levels of eotaxin-3, periostin, thymus and activation-regulated chemokine (TARC), and total IgE, may serve as markers to track the effectiveness of dupilumab treatment [99,100]. Regardless of blood eosinophils or FeNO, 1902 asthma patients who experienced one or more exacerbations in the previous 12 months were recruited for the LIBERTY ASTHMA QUEST trial [98]. Only individuals with a blood eosinophil level of ≥300 cells/μL showed a statistically significant advantage, as their AER decreased by almost two-thirds. Individuals with a FeNO ≥ 25 ppb also exhibited clinical effectiveness. Additionally, dupilumab is effective in reducing the need for oral corticosteroids. Compared to placebo, the LIBERTY ASTHMA VENTURE study showed a 50% overall median decrease in prednisolone dose [101]. Regarding comorbidities, when compared to placebo, dupilumab therapy for individuals with moderate-to-severe atopic dermatitis improved subjective symptoms, quality of life scores, sadness, and anxiety scores in addition to objective disease indicators [97]. Thus, for patients with moderate-to-severe asthma and atopic dermatitis, dupilumab may be preferred as the first choice rather than other biologic drugs. Furthermore, dupilumab has received approval for nasal polyposis, eosinophilic esophagitis, and eosinophilic COPD [102].

### 4.6. Tezepelumab

Tezepelumab is a fully human monoclonal antibody directed against thymic stromal lymphopoietin (TSLP), an essential epithelial alarmin. It is administered subcutaneously every 28 days at a dose of 210 mg. Responding to several noxious agents, airway epithelial cells release TSLP, which is involved in both T2 and non-T2 (T2 low) asthma [103]. Indeed, by stimulating dendritic cells and promoting Th17 polarization, TSLP has also been linked to the development of neutrophilic inflammation [104]. By targeting an upstream epithelial alarmin rather than downstream pathways, tezepelumab may halt the inflammatory process associated with asthma, distinguishing it from previous biologics [105]. Some studies have assessed the efficacy of this biological drug. The NAVIGATOR study was a phase 3 randomized controlled trial [106]. Inclusion requirements included two or more severe exacerbations during the past 12 months, at least a moderate dose of ICS, and an additional controller drug. Compared to placebo, tezepelumab therapy resulted in a 56% reduction in exacerbation rates. Those with the highest T2 signatures (greater blood eosinophils and higher FeNO) showed the most significant decreases. However, even patients with blood eosinophil counts below 150 cells/μL benefited significantly from treatment with tezepelumab (RR of 0.47) if their FeNO was at least 25 ppb. Only the dual biomarker low group (blood eosinophils < 150 cells/μL and low FeNO < 25 ppb) exhibited no effectiveness. The PATHWAY trial was the first clinical research to demonstrate the efficacy of tezepelumab in treating patients with severe, uncontrolled asthma [107]. The primary outcome at week 52 was the annualized rate of asthma flare-ups. This study demonstrated that tezepelumab resulted in statistically significant reductions in the exacerbation rate at week 52. Furthermore, a post-hoc analysis of the same PATHWAY study indicated that tezepelumab decreased the exacerbation rate in patients with nasal polyps more than in those without nasal polyps [108]. The UPSTREAM trial was a phase 2, double-blind, placebo-controlled, randomized investigation that evaluated intravenous tezepelumab 700 mg or placebo every four weeks for a total of 12 weeks, focusing on adult patients with asthma and airway hyperresponsiveness to mannitol [109]. This study demonstrated that, as evidenced by a decrease in airway hyperresponsiveness to mannitol, blocking TSLP may have benefits beyond reducing type 2 airway inflammation. Patients with OCS-dependent asthma were recruited for the SOURCE study and randomly assigned to receive either tezepelumab or a placebo [110]. The results showed that the likelihood of achieving a greater percentage reduction in OCS maintenance dose at week 48 was higher in the tezepelumab group compared to the placebo group. Lastly, the DESTINATION study was the first extended, long-term biological treatment trial for severe asthma [111]. The objective was to compare the safety and tolerability of tezepelumab against placebo over a year.

### 4.7. Head-to-Head Comparison Among Biologicals

Shortly, there is a need for data on the comparative effectiveness of biologics. Due to the lack of existing head-to-head trials, this will necessitate a combination of real-world evidence, systematic reviews, network meta-analyses, and direct head-to-head randomized comparisons of biologics, including the ongoing PREDICTUMAB study [ClinicalTrials.gov identifier: NCT03476109]. To optimize the therapeutic response to biologic treatments, implementing a precise process for accurate diagnosis and phenotyping/endotyping of severe type 2 asthma is essential, aiming to select the most suitable drug for each patient. The absence of direct comparative studies among existing anti-asthma biologics hinders the establishment of a trustworthy efficacy hierarchy. Nonetheless, recent systematic reviews and network meta-analyses of randomized controlled trials (RCTs) have offered valuable insights. Specifically, dupilumab and tezepelumab stand out as the most effective biologics for reducing asthma exacerbations and enhancing FEV_1_, while anti-IL-5/IL-5 receptor antibodies tend to excel as drugs that spare oral corticosteroids [112].

Eventual head-to-head comparisons should also refer to the relevance of comorbidities. Indeed, we should be aware of the added therapeutic value which some comorbidities offer to patients with severe asthma. In particular, when nasal polyps occur together with severe asthma, the latter is characterized by better therapeutic responses to biologics which are indicated for both these comorbidities.

## 5. Decision-Making Process

**Figure 1:**
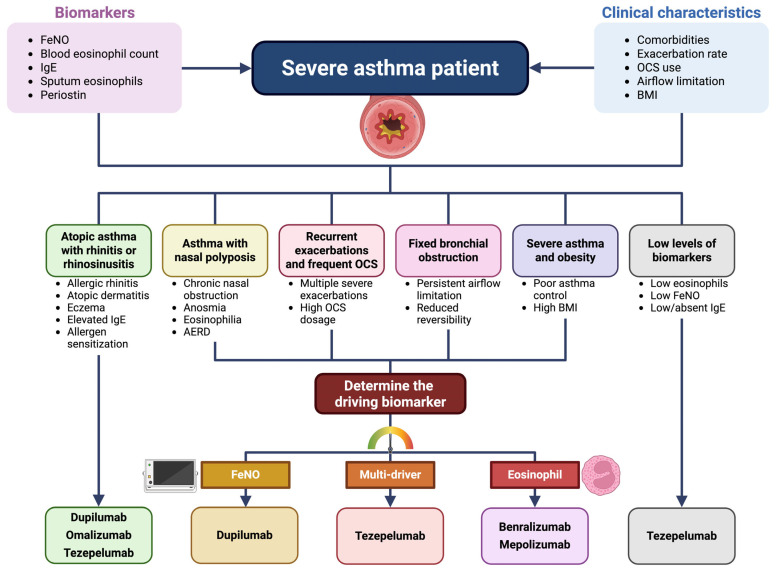
Decision-making process for selecting the most appropriate biologic therapy in severe asthma. Abbreviations: FeNO, fractional exhaled nitric oxide; IgE, immunoglobulins E; OCS, oral corticosteroids; BMI, body mass index; AERD, aspirin-exacerbated respiratory disease. This original figure was created by the authors using “BioRender.com” https://biorender.com (accessed on 24 May 2025).

The decision-making process for selecting the most appropriate biologic therapy in severe asthma is multifaceted. It should be based on a combination of clinical judgment, biomarker evaluation, and patient-specific factors. The first step involves a comprehensive clinical assessment to confirm the diagnosis of severe asthma, which is typically characterized by persistent symptoms, frequent exacerbations, and the need for high-dose inhaled corticosteroids plus additional controller medications, often including recurrent OCS courses (Figure 1).

The selection of the most appropriate biologic therapy in severe asthma is inherently multifactorial, requiring a holistic, personalized approach that integrates clinical phenotyping, biomarker analysis, comorbidity assessment, and consideration of patient preferences and treatment feasibility. This complex clinical decision-making strategy should commence with a thorough evaluation of asthma severity based on established criteria, including persistent respiratory symptoms, frequent exacerbations requiring systemic corticosteroids, and dependence on high-dose inhaled corticosteroids combined with long-acting bronchodilators. Once the diagnosis of severe asthma is established, the next step is to identify the underlying inflammatory endotype. This requires evaluating key biomarkers such as blood eosinophil counts (typically ≥ 150 cells/μL for treatment initiation and ≥300 cells/μL as a marker of sustained eosinophilic inflammation), fractional exhaled nitric oxide (FeNO) levels (≥25 ppb as an indicator of IL-13-driven inflammation), and total or specific IgE levels in the context of allergic sensitization to environmental aeroallergens. These biomarkers not only help to classify patients as having T2-high or T2-low inflammation, but also assist in predicting the therapeutic response to specific biologic agents.

Following confirmation of severity, clinicians should classify patients based on clinical phenotypes and inflammatory endotypes. For patients with an apparent allergic phenotype, characterized by elevated IgE and coexisting conditions such as allergic rhinitis or allergic conjunctivitis, omalizumab is often the preferred option. This anti-IgE monoclonal antibody is particularly effective in reducing exacerbations in patients with allergen-driven disease. The selection of omalizumab is further supported by skin prick testing or in vitro measurement of allergen-specific IgE.

Patients with a history of nasal polyposis or aspirin-exacerbated respiratory disease (AERD) typically exhibit eosinophilic inflammation. In these patients, blood eosinophil counts above 300 cells/μL and elevated FeNO levels support the selection of anti-IL-5 (mepolizumab, reslizumab) or anti-IL-5 receptor (benralizumab) therapies. Dupilumab, targeting IL-4 and IL-13 receptors, is also effective in patients with nasal polyposis, particularly those with coexisting elevated FeNO or atopic dermatitis.

Frequent exacerbators with persistent OCS dependence represent a high-priority group for biologic treatment. These patients may benefit from any of the currently available biologics depending on their biomarker profiles, with strong evidence supporting mepolizumab, benralizumab, and dupilumab in reducing exacerbation rates and minimizing OCS requirements. Anyway, partially unsuccessful biologic therapies, characterized by the persistence of patient’s unmet needs, may require a shift towards another monoclonal antibody.

For patients presenting with fixed bronchial obstruction, typically characterized by irreversible airflow limitation on spirometry, biologics targeting eosinophilic inflammation may still provide benefit if the blood eosinophil count is according to the prescription indications. However, structural airway remodeling may limit reversibility, suggesting realistic patient expectations regarding treatment outcomes. Obesity-associated asthma represents a clinical phenotype often marked by low T2 inflammation and mechanical or metabolic influences. These patients may exhibit low eosinophil counts and normal FeNO despite a high symptom burden. By targeting upstream TSLP, tezepelumab is beneficial in such a difficult-to-treat group, and this biologic has been approved regardless of biomarker status.

Finally, patients with low or non-specific biomarker profiles, but who experience persistent symptoms and exacerbations, pose a therapeutic challenge. Tezepelumab again represents the biologic of choice here due to its efficacy in patients with low biomarkers and a broad range of inflammatory patterns. In such cases, it is essential to reassess for non-adherence, incorrect inhaler technique, or undiagnosed comorbidities such as vocal cord dysfunction, bronchiectasis, or psychological disorders.

Importantly, treatment selection should also account for practical considerations, including dosing frequency, route of administration (subcutaneous or intravenous), self-administration capability, patient preference, and availability of healthcare resources. The expected duration of therapy should be discussed with the patient, and treatment response should be assessed after 4 to 12 months based on symptom control, exacerbation frequency, OCS sparing, lung function, and quality of life. If the treatment response is suboptimal, clinicians should ensure that the patient is adherent to background inhaled therapy, reassess environmental and lifestyle factors, and consider switching to an alternative biologic with a different mechanism of action. This iterative, personalized treatment approach maximizes the likelihood of achieving optimal disease control, reducing the burden of exacerbations, and improving long-term patient outcomes. The selection of the most appropriate biologic therapy for patients with severe asthma should be firmly grounded on the recognition and analysis of key clinical characteristics and biomarker profiles. This individualized strategy demands a detailed understanding of each patient’s disease phenotype, comorbidities, and inflammatory endotype. Overall, biologic therapy should be selected based on an integrated profile that combines clinical characteristics and biomarker results. Beyond biomarker thresholds, attention must be given to the temporal dynamics of disease (onset, course), comorbidity clustering, exacerbation triggers, and therapeutic preferences. Treatment success is best ensured by early intervention, objective reassessment after 4–12 months, and ongoing optimization of adjunct therapies and modifiable factors. The therapeutic decision-making process in severe asthma relies upon a precise evaluation of clinical phenotypes and endotypes, with particular emphasis on the constellation of clinical features, comorbid conditions, and biomarker profiles that characterize each patient. An accurate and deep understanding of these clinical traits enables a tailored selection of biologic therapy, aimed at optimizing disease control, minimizing exacerbations, and improving the patient’s overall quality of life. Personalizing the choice of biologic treatment for severe asthma represents a cornerstone of modern respiratory care. The decision-making process must integrate clinical phenotyping, biomarker stratification, and comorbidity profiling, ensuring that treatment is matched to the unique inflammatory drivers and clinical presentation of each patient. This section provides a stepwise, highly detailed evaluation of the various clinical scenarios that clinicians commonly encounter.

A further relevant aspect which should be considered when choosing a monoclonal antibody for add-on treatment of severe asthma refers to the rapidity of the therapeutic effects. Indeed, with respect to slowly acting omalizumab, modern biologics (i.e., mepolizumab, benralizumab, dupilumab, tezepelumab) are characterized by very early clinical and functional responses [113,114,115]. The latter translate into a better quality of life, and also offer to severe asthmatic patients a satisfied perception of their overall improvements.

In summary, optimal biologic selection requires an integrated, dynamic, and patient-centered approach that leverages clinical expertise, objective assessment tools, and shared decision-making to deliver precision care in managing severe asthma. These detailed, phenotype-specific recommendations provide clinicians with a structured framework for selecting biologics tailored to individual patient needs, optimizing outcomes in the management of severe asthma. In real-world clinical practice, a good therapeutic response to biologic drugs used for treatment of severe asthma can be also associated with important economic advantages in countries characterized by a wide coverage provided by national health systems. Indeed, despite the high costs of these monoclonal antibodies, their long-term use makes it possible to significantly lower hospitalizations for severe asthma exacerbations, as well as accesses to emergency room, intensive care units, and unscheduled medical visits. Such a relevant decrement of direct costs can be coupled with further indirect benefits including a decrease of lost working days, a better scholastic performance, and a significant improvement of subjective depression and anxiety.

## 6. Conclusions

Considering the various presentations and characteristics of severe asthma, the procedure for selecting the most suitable biological treatment should be guided by the patient’s clinical presentation and preferences, starting from the phenotype and presumed endotype. We can certainly utilize the well-known biomarkers, which include serum total IgE, allergen-specific IgE, blood eosinophil count, and FeNO. When patients are taking high dosages of ICS, current guidelines suggest evaluating the inflammatory phenotype. Before characterizing asthma phenotype, it is advisable to eliminate or at least reduce the dose of OCS. Additionally, we should investigate the patient’s history of exacerbations, current use of OCS, and comorbidities (including atopic dermatitis, chronic rhinosinusitis, and eosinophilic granulomatosis with polyangiitis). We should also consider additional aspects, such as the interval between doses, the administration route, and the potential for self-injection, as well as the need to adjust dosages based on body weight. Despite receiving biologic treatment, a small percentage of patients still experience poor asthma control, including frequent exacerbations, even though the medication efficacy in treating severe asthma is usually satisfactory. According to new recommendations, biologics should be used for at least four months before assessing the response. In cases of unclear response, we can extend this period to 6 or 12 months. It is also essential to ascertain any exposure to smoking or other environmental factors, as well as to evaluate whether the patient adheres to the basic inhalation therapy. Hopefully, more treatment options for people with severe asthma will be developed in the next future, powered by the progressive advances in the elucidation of the cellular and molecular mechanisms underlying this complex and heterogeneous disease.

## Figures and Tables

**Table 1:** Characteristics and clinical utility of biomarkers for severe asthma management.

Biomarker	Key Pathway	Cut-Off	Diagnostic Accuracy	Clinical Utility and Practical Considerations
**FeNO**	IL-13, iNOS 2	Adults: <25 ppb (low), 25–50 ppb (intermediate), >50 ppb (high) Children: <20 ppb (low), 20–35 ppb (intermediate), >35 ppb (high)	Sensitivity: 0.70–0.80 Specificity: 0.60–0.70	Non-invasive surrogate of eosinophilic airway inflammation; useful for identifying T2-high asthma, predicting corticosteroid response, and monitoring effectiveness/adherence in ICS-treated patients. Useful guide for anti-IL-4/13R therapy
**Blood eosinophil count**	IL-5	≥150 cells/µL (T2-high indicator) ≥300 cells/µL (higher risk/response)	Sensitivity: 0.60–0.75 Specificity: 0.70–0.85	Widely available and inexpensive; supports diagnosis of eosinophilic asthma. Used to guide biologic therapy (e.g., anti-IL-5/IL-5R). Influenced by infections, diurnal variation, and steroid use
**Sputum eosinophils**	Direct measure of airway eosinophilic inflammation	>2–3% of non-squamous cells	Sensitivity: 0.80–0.90 Specificity: 0.80–0.90	Considered the gold standard for detecting airway eosinophilia; useful for personalized corticosteroid titration. Limited by need for laboratory processing and poor availability in routine practice
**Serum IgE**	IL-4/IL-13 pathway (antibody class switching)	>100 IU/mL often considered elevated Positive specific IgE	Sensitivity: 0.40–0.60 Specificity: 0.50–0.70	Marker of atopic phenotype; used primarily to assess eligibility for anti-IgE therapy. Poor correlation with disease severity and eosinophilic inflammation. Influenced by multiple factors including age and comorbidities
**Serum periostin**	IL-13 pathway, Tissue remodeling (TGF-β)	No standardized cut-off	Sensitivity: 0.60–0.75 Specificity: 0.70–0.85	Reflects IL-13 activity. Limited availability and poor assay standardization hinder clinical application

Abbreviations. FeNO, Fractional exhaled nitric oxide; iNOS 2, Inducible nitric oxide synthase 2; ICS, Inhaled corticosteroids; TGF-β, Transforming growth factor-β.

**Table 2:** Biologic agents available for severe asthma treatment.

Biologic Agent	Target	Indications	Biomarkers	Age	Dosing Regimen	Clinical Trials
**Omalizumab**	IgE	Severe allergic asthma Chronic idiopathic urticaria CRSwNP Food allergy	IgE between 30–1500 UI/mL	≥6 years	Dependent on patient’s weight and serum IgE levels, s.c.	INNOVATE NCT00314574 NCT00377572 EXTRA ICATA
**Mepolizumab**	IL-5	Severe eosinophilic asthma EGPA HES CRSwNP COPD	Eos ≥ 150 cells/µL before first administration or Eos ≥ 300 cells/µL in the previous year	≥6 years	100 mg s.c. every 28 days for adults 40 mg s.c. every 28 days for children	DREAM MENSA SIRIUS MUSCA COSMOS
**Reslizumab**	IL-5	Severe eosinophilic asthma	Eos ≥ 400 cells/μL	≥18 years	3 mg/kg every 28 days i.v.	NCT01287039 NCT01285323 NCT01508936
**Benralizumab**	IL-5R	Severe eosinophilic asthma EGPA	Eos ≥ 300 cells/µL	≥6 years	30 mg s.c. every 28 days for the first three administrations, then every 56 days	SIROCCO CALIMA ZONDA MANDARA
**Dupilumab**	IL-4R/ IL-13R	Severe type 2 asthma CRSwNP Atopic dermatitis EoE Prurigo nodularis COPD	Eos ≥ 150 cells/µL or FeNO ≥ 25 ppb	≥6 years	400 mg s.c. for the first dose, followed by one injection of 200 mg every 14 days or first dose of 600 mg sc, followed by 300 mg every 14 days	QUEST VENTURE TRAVERSE
**Tezepelumab**	TSLP	Severe asthma	—	≥12 years	210 mg s.c. every 28 days	NAVIGATOR PATHWAY UPSTREAM SOURCE DESTINATION CASCADE

Abbreviations. CRSwNP, Chronic rhinosinusitis with nasal polyp; EGPA, Eosinophilic granulomatosis with polyangiitis; HES, Hypereosinophilic syndrome; EoE, Eosinophilic esophagitis; COPD, Chronic obstructive pulmonary disease.

## Data Availability

Not applicable.

## References

[1] Chung K.F., Wenzel S.E., Brozek J.L., Bush A., Castro M., Sterk P.J., Adcock I.M., Bateman E.D., Bel E.H., Bleecker E.R. (2014). International ERS/ATS guidelines on definition, evaluation and treatment of severe asthma. Eur. Respir. J..

[2] Stern J., Pier J., Litonjua A.A. (2020). Asthma epidemiology and risk factors. Semin. Immunopathol..

[3] Papi A., Brightling C., Pedersen S.E., Reddel H.K. (2018). Asthma. Lancet.

[4] Kuruvilla M.E., Lee F.E., Lee G.B. (2019). Understanding Asthma Phenotypes, Endotypes, and Mechanisms of Disease. Clin. Rev. Allergy Immunol..

[5] Lugogo N.L., Akuthota P. (2021). Type 2 Biomarkers in Asthma: Yet Another Reflection of Heterogeneity. J. Allergy Clin. Immunol. Pract..

[6] Choy D.F., Hart K.M., Borthwick L.A., Shikotra A., Nagarkar D.R., Siddiqui S., Jia G., Ohri C.M., Doran E., Vannella K.M. (2015). TH2 and TH17 inflammatory pathways are reciprocally regulated in asthma. Sci. Transl. Med..

[7] Murugesan N., Saxena D., Dileep A., Adrish M., Hanania N.A. (2023). Update on the Role of FeNO in Asthma Management. Diagnostics.

[8] Menzies-Gow A., Mansur A.H., Brightling C.E. (2020). Clinical utility of fractional exhaled nitric oxide in severe asthma management. Eur. Respir. J..

[9] Dweik R.A., Boggs P.B., Erzurum S.C., Irvin C.G., Leigh M.W., Lundberg J.O., Olin A.C., Plummer A.L., Taylor D.R., American Thoracic Society Committee on Interpretation of Exhaled Nitric Oxide Levels (FENO) for Clinical Applications (2011). An official ATS clinical practice guideline: Interpretation of exhaled nitric oxide levels (FENO) for clinical applications. Am. J. Respir. Crit. Care Med..

[10] Escamilla-Gil J.M., Fernandez-Nieto M., Acevedo N. (2022). Understanding the Cellular Sources of the Fractional Exhaled Nitric Oxide (FeNO) and Its Role as a Biomarker of Type 2 Inflammation in Asthma. Biomed. Res. Int..

[11] Heffler E., Carpagnano G.E., Favero E., Guida G., Maniscalco M., Motta A., Paoletti G., Rolla G., Baraldi E., Pezzella V. (2020). Fractional Exhaled Nitric Oxide (FENO) in the management of asthma: A position paper of the Italian Respiratory Society (SIP/IRS) and Italian Society of Allergy, Asthma and Clinical Immunology (SIAAIC). Multidiscip. Respir. Med..

[12] Bonini M., Annibale R., Barbaglia S., Bo M., Capano F., Celeste M., Di Girolamo Faraone P., Ferri S., Galeone C., Picozza M. (2025). The role of Fraction Exhaled Nitric Oxide (FeNO) in asthma management: An Italian consensus statement on clinical and economic aspects. Multidiscip. Respir. Med..

[13] Maniscalco M., Candia C., Visca D., D’Amato M., Calabrese C., Ambrosino P., Molino A., Fuschillo S. (2024). Revealing the gap: Fractional exhaled nitric oxide and clinical responsiveness to biological therapy in severe asthma—A retrospective study. ERJ Open Res..

[14] Soendergaard M.B., Hansen S., Håkansson K.E.J., von Bülow A., Bjerrum A.S., Schmid J.M., Johansson S.L., Rasmussen L.M., Johnsen C.R., Bertelsen B.B. (2025). Early Reduction of FeNO on Anti-IL5 Biologics Is Associated With Clinical Remission of Severe Asthma. Allergy.

[15] Pavord I.D., Deniz Y., Corren J., Casale T.B., FitzGerald J.M., Izuhara K., Daizadeh N., Ortiz B., Johnson R.R., Harel S. (2023). Baseline FeNO Independently Predicts the Dupilumab Response in Patients with Moderate-to-Severe Asthma. J. Allergy Clin. Immunol. Pract..

[16] Busse W.W., Wenzel S.E., Casale T.B., FitzGerald J.M., Rice M.S., Daizadeh N., Deniz Y., Patel N., Harel S., Rowe P.J. (2021). Baseline FeNO as a prognostic biomarker for subsequent severe asthma exacerbations in patients with uncontrolled, moderate-to-severe asthma receiving placebo in the LIBERTY ASTHMA QUEST study: A post-hoc analysis. Lancet Respir. Med..

[17] Menzies-Gow A., Ambrose C.S., Colice G., Hunter G., Cook B., Molfino N.A., Llanos J.P., Israel E. (2023). Effect of Tezepelumab on Lung Function in Patients With Severe, Uncontrolled Asthma in the Phase 3 NAVIGATOR Study. Adv. Ther..

[18] Castillo J.R., Peters S.P., Busse W.W. (2017). Asthma Exacerbations: Pathogenesis, Prevention, and Treatment. J. Allergy Clin. Immunol. Pract..

[19] Janson C., Bjermer L., Lehtimäki L., Kankaanranta H., Karjalainen J., Altraja A., Yasinska V., Aarli B., Rådinger M., Hellgren J. (2022). Eosinophilic airway diseases: Basic science, clinical manifestations and future challenges. Eur. Clin. Respir. J..

[20] Bai C., Jiang D., Wang L., Xue F., Chen O. (2019). A high blood eosinophil count may be a risk factor for incident asthma in population at risk. Respir. Med..

[21] Bleecker E.R., Meyers D.A., Billheimer D., Li H., Newbold P., Kwiatek J., Hirsch I., Katial R., Li X. (2023). Clinical Implications of Longitudinal Blood Eosinophil Counts in Patients With Severe Asthma. J. Allergy Clin. Immunol. Pract..

[22] Schoettler N., Strek M.E. (2020). Recent Advances in Severe Asthma: From Phenotypes to Personalized Medicine. Chest.

[23] Frøssing L., Silberbrandt A., Von Bülow A., Backer V., Porsbjerg C. (2021). The Prevalence of Subtypes of Type 2 Inflammation in an Unselected Population of Patients with Severe Asthma. J. Allergy Clin. Immunol. Pract..

[24] Ojanguren I., Quirce S., Bobolea I., Pérez de Llano L., Del Pozo V. (2025). Phenotyping Asthma Exacerbations: One Step Further in the Management of Severe Asthma. J. Investig. Allergol. Clin. Immunol..

[25] Hussain M., Liu G. (2024). Eosinophilic Asthma: Pathophysiology and Therapeutic Horizons. Cells.

[26] García-Moguel I., Martínez-Mesa Á., Andújar-Espinosa R., Díaz-Campos R., Velasco-Garrido J.L., Sanchez-Trincado J.L., Luzon E., Nuevo J., Alconada C., Gutiérrez M.Á. (2025). The impact of blood eosinophil count and FeNO on benralizumab effectiveness in clinical practice: An ORBE II subanalysis. Respir. Med..

[27] Izuhara K., Conway S.J., Moore B.B., Matsumoto H., Holweg C.T., Matthews J.G., Arron J.R. (2016). Roles of Periostin in Respiratory Disorders. Am. J. Respir. Crit. Care Med..

[28] Joseph C., Tatler A.L. (2022). Pathobiology of Airway Remodeling in Asthma: The Emerging Role of Integrins. J. Asthma Allergy.

[29] Fouka E., Domvri K., Gkakou F., Alevizaki M., Steiropoulos P., Papakosta D., Porpodis K. (2022). Recent insights in the role of biomarkers in severe asthma management. Front. Med..

[30] Pelaia C., Pelaia G., Crimi C., Maglio A., Armentaro G., Calabrese C., Sciacqua A., Gallelli L., Vatrella A. (2022). Biological Therapy of Severe Asthma with Dupilumab, a Dual Receptor Antagonist of Interleukins 4 and 13. Vaccines.

[31] Yancey S.W., Keene O.N., Albers F.C., Ortega H., Bates S., Bleecker E.R., Pavord I. (2017). Biomarkers for severe eosinophilic asthma. J. Allergy Clin. Immunol..

[32] Guida G., Bagnasco D., Carriero V., Bertolini F., Ricciardolo F.L.M., Nicola S., Brussino L., Nappi E., Paoletti G., Canonica G.W. (2022). Critical evaluation of asthma biomarkers in clinical practice. Front. Med..

[33] Zeiger R.S., Schatz M., Dalal A.A., Chen W., Sadikova E., Suruki R.Y., Kawatkar A.A., Qian L. (2017). Blood Eosinophil Count and Outcomes in Severe Uncontrolled Asthma: A Prospective Study. J. Allergy Clin. Immunol. Pract..

[34] Oppenheimer J., Hoyte F.C.L., Phipatanakul W., Silver J., Howarth P., Lugogo N.L. (2022). Allergic and eosinophilic asthma in the era of biomarkers and biologics: Similarities, differences and misconceptions. Ann. Allergy Asthma Immunol..

[35] Gevaert P., Wong K., Millette L.A., Carr T.F. (2022). The Role of IgE in Upper and Lower Airway Disease: More Than Just Allergy!. Clin. Rev. Allergy Immunol..

[36] Menzella F., Just J., Sauerbeck I.S., Mailaender C., Saccheri F., Thonnelier C., Jaumont X., Mala L. (2023). Omalizumab for the treatment of patients with severe allergic asthma with immunoglobulin E levels above >1500 IU/mL. World Allergy Organ. J..

[37] Meng J., Xiao H., Xu F., She X., Liu C., Canonica G.W. (2025). Systemic barrier dysfunction in type 2 inflammation diseases: Perspective in the skin, airways, and gastrointestinal tract. Immunol. Res..

[38] Caruso C., Colantuono S., Nicoletti A., Arasi S., Firinu D., Gasbarrini A., Coppola A., Di Michele L. (2021). Metabolomics, Microbiota, and In Vivo and In Vitro Biomarkers in Type 2 Severe Asthma: A Perspective Review. Metabolites.

[39] Humbert M., Bousquet J., Bachert C., Palomares O., Pfister P., Kottakis I., Jaumont X., Thomsen S.F., Papadopoulos N.G. (2019). IgE-Mediated Multimorbidities in Allergic Asthma and the Potential for Omalizumab Therapy. J. Allergy Clin. Immunol. Pract..

[40] Lambrecht B.N., Hammad H., Fahy J.V. (2019). The Cytokines of Asthma. Immunity.

[41] León B., Ballesteros-Tato A. (2021). Modulating Th2 Cell Immunity for the Treatment of Asthma. Front. Immunol..

[42] Chai W., Zhang X., Lin M., Chen Z., Wang X., Wang C., Chen A., Wang C., Wang H., Yue H. (2022). Allergic rhinitis, allergic contact dermatitis and disease comorbidity belong to separate entities with distinct composition of T-cell subsets, cytokines, immunoglobulins and autoantibodies. Allergy Asthma Clin. Immunol..

[43] Xie X., Xuan L., Zhao Y., Wang X., Zhang L. (2023). Diverse Endotypes of Chronic Rhinosinusitis and Clinical Implications. Clin. Rev. Allergy Immunol..

[44] Corren J. (1997). Allergic rhinitis and asthma: How important is the link?. J. Allergy Clin. Immunol..

[45] Stevens W.W., Peters A.T., Tan B.K., Klingler A.I., Poposki J.A., Hulse K.E., Grammer L.C., Welch K.C., Smith S.S., Conley D.B. (2019). Associations Between Inflammatory Endotypes and Clinical Presentations in Chronic Rhinosinusitis. J. Allergy Clin. Immunol. Pract..

[46] Georgopoulos R., Krouse J.H., Toskala E. (2014). Why otolaryngologists and asthma are a good match: The allergic rhinitis-asthma connection. Otolaryngol. Clin. N. Am..

[47] Klingler A.I., Stevens W.W., Tan B.K., Peters A.T., Poposki J.A., Grammer L.C., Welch K.C., Smith S.S., Conley D.B., Kern R.C. (2021). Mechanisms and biomarkers of inflammatory endotypes in chronic rhinosinusitis without nasal polyps. J. Allergy Clin. Immunol..

[48] Delemarre T., Holtappels G., De Ruyck N., Zhang N., Nauwynck H., Bachert C., Gevaert E. (2020). Type 2 inflammation in chronic rhinosinusitis without nasal polyps: Another relevant endotype. J. Allergy Clin. Immunol..

[49] Cunico D., Giannì G., Scavone S., Buono E.V., Caffarelli C. (2024). The Relationship Between Asthma and Food Allergies in Children. Children.

[50] Wood R.A., Togias A., Sicherer S.H., Shreffler W.G., Kim E.H., Jones S.M., Leung D.Y.M., Vickery B.P., Bird J.A., Spergel J.M. (2024). Omalizumab for the Treatment of Multiple Food Allergies. N. Engl. J. Med..

[51] Laidlaw T.M., Mullol J., Woessner K.M., Amin N., Mannent L.P. (2021). Chronic Rhinosinusitis with Nasal Polyps and Asthma. J. Allergy Clin. Immunol. Pract..

[52] Maspero J., Adir Y., Al-Ahmad M., Celis-Preciado C.A., Colodenco F.D., Giavina-Bianchi P., Lababidi H., Ledanois O., Mahoub B., Perng D.W. (2022). Type 2 inflammation in asthma and other airway diseases. ERJ Open Res..

[53] Rogers L., Jesenak M., Bjermer L., Hanania N.A., Seys S.F., Diamant Z. (2023). Biologics in severe asthma: A pragmatic approach for choosing the right treatment for the right patient. Respir. Med..

[54] Chung L.P., Upham J.W., Bardin P.G., Hew M. (2020). Rational oral corticosteroid use in adult severe asthma: A narrative review. Respirology.

[55] Cataldo D., Louis R., Michils A., Peché R., Pilette C., Schleich F., Ninane V., Hanon S. (2021). Severe asthma: Oral corticosteroid alternatives and the need for optimal referral pathways. J. Asthma.

[56] Sweeney J., Patterson C.C., Menzies-Gow A., Niven R.M., Mansur A.H., Bucknall C., Chaudhuri R., Price D., Brightling C.E., Heaney L.G. (2016). Comorbidity in severe asthma requiring systemic corticosteroid therapy: Cross-sectional data from the Optimum Patient Care Research Database and the British Thoracic Difficult Asthma Registry. Thorax.

[57] Varricchi G., Ferri S., Pepys J., Poto R., Spadaro G., Nappi E., Paoletti G., Virchow J.C., Heffler E., Canonica W.G. (2022). Biologics and airway remodeling in severe asthma. Allergy.

[58] Fish J.E., Peters S.P. (1999). Airway remodeling and persistent airway obstruction in asthma. J. Allergy Clin. Immunol..

[59] Pelaia G., Vatrella A., Maselli R. (2012). The potential of biologics for the treatment of asthma. Nat. Rev. Drug Discov..

[60] Tan R., Liew M.F., Lim H.F., Leung B.P., Wong W.S.F. (2020). Promises and challenges of biologics for severe asthma. Biochem. Pharmacol..

[61] Farne H.A., Wilson A., Milan S., Banchoff E., Yang F., Powell C.V. (2022). Anti-IL-5 therapies for asthma. Cochrane Database Syst. Rev..

[62] Agache I., Beltran J., Akdis C., Akdis M., Canelo-Aybar C., Canonica G.W., Casale T., Chivato T., Corren J., Del Giacco S. (2020). Efficacy and safety of treatment with biologicals (benralizumab, dupilumab, mepolizumab, omalizumab and reslizumab) for severe eosinophilic asthma. A systematic review for the EAACI Guidelines—Recommendations on the use of biologicals in severe asthma. Allergy.

[63] Castro M., Papi A., Porsbjerg C., Lugogo N.L., Brightling C.E., González-Barcala F.J., Bourdin A., Ostrovskyy M., Staevska M., Chou P.C. (2025). Effect of dupilumab on exhaled nitric oxide, mucus plugs, and functional respiratory imaging in patients with type 2 asthma (VESTIGE): A randomised, double-blind, placebo-controlled, phase 4 trial. Lancet Respir. Med..

[64] Peters U., Dixon A.E., Forno E. (2018). Obesity and asthma. J. Allergy Clin. Immunol..

[65] Sharma V., Cowan D.C. (2021). Obesity, Inflammation, and Severe Asthma: An Update. Curr. Allergy Asthma Rep..

[66] Corren J., Pham T.H., Garcia Gil E., Sałapa K., Ren P., Parnes J.R., Colice G., Griffiths J.M. (2022). Baseline type 2 biomarker levels and response to tezepelumab in severe asthma. Allergy.

[67] Corren J., Menzies-Gow A., Chupp G., Israel E., Korn S., Cook B., Ambrose C.S., Hellqvist Å., Roseti S.L., Molfino N.A. (2023). Efficacy of Tezepelumab in Severe, Uncontrolled Asthma: Pooled Analysis of the PATHWAY and NAVIGATOR Clinical Trials. Am. J. Respir. Crit. Care Med..

[68] Pelaia C., Pelaia G., Longhini F., Crimi C., Calabrese C., Gallelli L., Sciacqua A., Vatrella A. (2021). Monoclonal Antibodies Targeting Alarmins: A New Perspective for Biological Therapies of Severe Asthma. Biomedicines.

[69] Niessen N.M., Fricker M., McDonald V.M., Gibson P.G. (2022). T2-low: What do we know?: Past, present, and future of biologic therapies in noneosinophilic asthma. Ann. Allergy Asthma Immunol..

[70] Miranda C., Busacker A., Balzar S., Trudeau J., Wenzel S.E. (2004). Distinguishing severe asthma phenotypes: Role of age at onset and eosinophilic inflammation. J. Allergy Clin. Immunol..

[71] Porsbjerg C., Melén E., Lehtimäki L., Shaw D. (2023). Asthma. Lancet.

[72] Hanania N.A., Wenzel S., Rosén K., Hsieh H.J., Mosesova S., Choy D.F., Lal P., Arron J.R., Harris J.M., Busse W. (2013). Exploring the effects of omalizumab in allergic asthma: An analysis of biomarkers in the EXTRA study. Am. J. Respir. Crit. Care Med..

[73] Casale T.B., Luskin A.T., Busse W., Zeiger R.S., Trzaskoma B., Yang M., Griffin N.M., Chipps B.E. (2019). Omalizumab Effectiveness by Biomarker Status in Patients with Asthma: Evidence From PROSPERO, A Prospective Real-World Study. J. Allergy Clin. Immunol. Pract..

[74] Pelaia C., Calabrese C., Barbuto S., Busceti M.T., Preianò M., Gallelli L., Savino R., Vatrella A., Pelaia G. (2019). Omalizumab lowers asthma exacerbations, oral corticosteroid intake and blood eosinophils: Results of a 5-YEAR single-centre observational study. Pulm. Pharmacol. Ther..

[75] Menzella F., Fontana M., Contoli M., Ruggiero P., Galeone C., Capobelli S., Simonazzi A., Catellani C., Scelfo C., Castagnetti C. (2022). Efficacy and Safety of Omalizumab Treatment Over a 16-Year Follow-Up: When a Clinical Trial Meets Real-Life. J. Asthma Allergy.

[76] Flood-Page P.T., Menzies-Gow A.N., Kay A.B., Robinson D.S. (2003). Eosinophil’s role remains uncertain as anti-interleukin-5 only partially depletes numbers in asthmatic airway. Am. J. Respir. Crit. Care Med..

[77] Shaker M., Briggs A., Dbouk A., Dutille E., Oppenheimer J., Greenhawt M. (2020). Estimation of Health and Economic Benefits of Clinic Versus Home Administration of Omalizumab and Mepolizumab. J. Allergy Clin. Immunol. Pract..

[78] Miyokawa R., Kivler C., Louie S., Godor D., Tan L., Kenyon N. (2020). Self-Administered Mepolizumab in the Management of Severe Asthma: Usability and Patient Acceptance. Patient Prefer. Adherence.

[79] Ortega H.G., Liu M.C., Pavord I.D., Brusselle G.G., FitzGerald J.M., Chetta A., Humbert M., Katz L.E., Keene O.N., Yancey S.W. (2014). Mepolizumab treatment in patients with severe eosinophilic asthma. N. Engl. J. Med..

[80] Bel E.H., Wenzel S.E., Thompson P.J., Prazma C.M., Keene O.N., Yancey S.W., Ortega H.G., Pavord I.D., SIRIUS Investigators (2014). Oral glucocorticoid-sparing effect of mepolizumab in eosinophilic asthma. N. Engl. J. Med..

[81] Wechsler M.E., Akuthota P., Jayne D., Khoury P., Klion A., Langford C.A., Merkel P.A., Moosig F., Specks U., Cid M.C. (2017). Mepolizumab or Placebo for Eosinophilic Granulomatosis with Polyangiitis. N. Engl. J. Med..

[82] Han J.K., Bachert C., Fokkens W., Desrosiers M., Wagenmann M., Lee S.E., Smith S.G., Martin N., Mayer B., Yancey S.W. (2021). Mepolizumab for chronic rhinosinusitis with nasal polyps (SYNAPSE): A randomised, double-blind, placebo-controlled, phase 3 trial. Lancet Respir. Med..

[83] Harrison T., Canonica G.W., Chupp G., Lee J., Schleich F., Welte T., Valero A., Gemzoe K., Maxwell A., Joksaite S. (2020). Real-world mepolizumab in the prospective severe asthma REALITI-A study: Initial analysis. Eur. Respir. J..

[84] Domvri K., Tsiouprou I., Bakakos P., Steiropoulos P., Katsoulis K., Kostikas K., Antoniou K.M., Papaioannou A.I., Rovina N., Katsaounou P. (2025). Effect of mepolizumab in airway remodeling in patients with late-onset severe asthma with an eosinophilic phenotype. J. Allergy Clin. Immunol..

[85] Corren J., Weinstein S., Janka L., Zangrilli J., Garin M. (2016). Phase 3 Study of Reslizumab in Patients With Poorly Controlled Asthma: Effects Across a Broad Range of Eosinophil Counts. Chest.

[86] Bernstein J.A., Virchow J.C., Murphy K., Maspero J.F., Jacobs J., Adir Y., Humbert M., Castro M., Marsteller D.A., McElhattan J. (2020). Effect of fixed-dose subcutaneous reslizumab on asthma exacerbations in patients with severe uncontrolled asthma and corticosteroid sparing in patients with oral corticosteroid-dependent asthma: Results from two phase 3, randomised, double-blind, placebo-controlled trials. Lancet Respir. Med..

[87] Manka L.A., Guntur V.P., Denson J.L., Dunn R.M., Dollin Y.T., Strand M.J., Wechsler M.E. (2021). Efficacy and safety of reslizumab in the treatment of eosinophilic granulomatosis with polyangiitis. Ann. Allergy Asthma Immunol..

[88] Kolbeck R., Kozhich A., Koike M., Peng L., Andersson C.K., Damschroder M.M., Reed J.L., Woods R., Dall’acqua W.W., Stephens G.L. (2010). MEDI-563, a humanized anti-IL-5 receptor alpha mAb with enhanced antibody-dependent cell-mediated cytotoxicity function. J. Allergy Clin. Immunol..

[89] Kankaanranta H., Ilmarinen P., Zhang X., Adcock I.M., Lahti A., Barnes P.J., Giembycz M.A., Lindsay M.A., Moilanen E. (2014). Tumour necrosis factor-α regulates human eosinophil apoptosis via ligation of TNF-receptor 1 and balance between NF-κB and AP-1. PLoS ONE.

[90] Louis R., Harrison T.W., Chanez P., Menzella F., Philteos G., Cosio B.G., Lugogo N.L., de Luiz G., Burden A., Adlington T. (2023). Severe Asthma Standard-of-Care Background Medication Reduction With Benralizumab: ANDHI in Practice Substudy. J. Allergy Clin. Immunol. Pract..

[91] Menzies-Gow A., Hoyte F.L., Price D.B., Cohen D., Barker P., Kreindler J., Jison M., Brooks C.L., Papeleu P., Katial R. (2022). Clinical Remission in Severe Asthma: A Pooled Post Hoc Analysis of the Patient Journey with Benralizumab. Adv. Ther..

[92] Bleecker E.R., FitzGerald J.M., Chanez P., Papi A., Weinstein S.F., Barker P., Sproule S., Gilmartin G., Aurivillius M., Werkström V. (2016). Efficacy and safety of benralizumab for patients with severe asthma uncontrolled with high-dosage inhaled corticosteroids and long-acting β2-agonists (SIROCCO): A randomised, multicentre, placebo-controlled phase 3 trial. Lancet.

[93] FitzGerald J.M., Bleecker E.R., Nair P., Korn S., Ohta K., Lommatzsch M., Ferguson G.T., Busse W.W., Barker P., Sproule S. (2016). Benralizumab, an anti-interleukin-5 receptor α monoclonal antibody, as add-on treatment for patients with severe, uncontrolled, eosinophilic asthma (CALIMA): A randomised, double-blind, placebo-controlled phase 3 trial. Lancet.

[94] Nair P., Wenzel S., Rabe K.F., Bourdin A., Lugogo N.L., Kuna P., Barker P., Sproule S., Ponnarambil S., Goldman M. (2017). Oral Glucocorticoid-Sparing Effect of Benralizumab in Severe Asthma. N. Engl. J. Med..

[95] Kavanagh J.E., Hearn A.P., Dhariwal J., d’Ancona G., Douiri A., Roxas C., Fernandes M., Green L., Thomson L., Nanzer A.M. (2021). Real-World Effectiveness of Benralizumab in Severe Eosinophilic Asthma. Chest.

[96] Pelaia C., Benfante A., Busceti M.T., Caiaffa M.F., Campisi R., Carpagnano G.E., Crimi N., D’Amato M., Foschino Barbaro M.P., Maglio A. (2023). Real-life effects of dupilumab in patients with severe type 2 asthma, according to atopic trait and presence of chronic rhinosinusitis with nasal polyps. Front. Immunol..

[97] Simpson E.L., Bieber T., Guttman-Yassky E., Beck L.A., Blauvelt A., Cork M.J., Silverberg J.I., Deleuran M., Kataoka Y., Lacour J.P. (2016). Two Phase 3 Trials of Dupilumab versus Placebo in Atopic Dermatitis. N. Engl. J. Med..

[98] Blauvelt A., de Bruin-Weller M., Gooderham M., Cather J.C., Weisman J., Pariser D., Simpson E.L., Papp K.A., Hong H.C., Rubel D. (2017). Long-term management of moderate-to-severe atopic dermatitis with dupilumab and concomitant topical corticosteroids (LIBERTY AD CHRONOS): A 1-year, randomised, double-blinded, placebo-controlled, phase 3 trial. Lancet.

[99] Wenzel S., Castro M., Corren J., Maspero J., Wang L., Zhang B., Pirozzi G., Sutherland E.R., Evans R.R., Joish V.N. (2016). Dupilumab efficacy and safety in adults with uncontrolled persistent asthma despite use of medium-to-high-dose inhaled corticosteroids plus a long-acting β2 agonist: A randomised double-blind placebo-controlled pivotal phase 2b dose-ranging trial. Lancet.

[100] Huang J., Pansare M. (2019). New Treatments for Asthma. Pediatr. Clin. N. Am..

[101] Rabe K.F., Nair P., Brusselle G., Maspero J.F., Castro M., Sher L., Zhu H., Hamilton J.D., Swanson B.N., Khan A. (2018). Efficacy and Safety of Dupilumab in Glucocorticoid-Dependent Severe Asthma. N. Engl. J. Med..

[102] Bhatt S.P., Rabe K.F., Hanania N.A., Vogelmeier C.F., Cole J., Bafadhel M., Christenson S.A., Papi A., Singh D., Laws E. (2023). Dupilumab for COPD with Type 2 Inflammation Indicated by Eosinophil Counts. N. Engl. J. Med..

[103] Ziegler S.F., Roan F., Bell B.D., Stoklasek T.A., Kitajima M., Han H. (2013). The biology of thymic stromal lymphopoietin (TSLP). Adv. Pharmacol..

[104] Tanaka J., Watanabe N., Kido M., Saga K., Akamatsu T., Nishio A., Chiba T. (2009). Human TSLP and TLR3 ligands promote differentiation of Th17 cells with a central memory phenotype under Th2-polarizing conditions. Clin. Exp. Allergy.

[105] Gauvreau G.M., Sehmi R., Ambrose C.S., Griffiths J.M. (2020). Thymic stromal lymphopoietin: Its role and potential as a therapeutic target in asthma. Expert. Opin. Ther. Targets.

[106] Menzies-Gow A., Colice G., Griffiths J.M., Almqvist G., Ponnarambil S., Kaur P., Ruberto G., Bowen K., Hellqvist Å., Mo M. (2020). NAVIGATOR: A phase 3 multicentre, randomized, double-blind, placebo-controlled, parallel-group trial to evaluate the efficacy and safety of tezepelumab in adults and adolescents with severe, uncontrolled asthma. Respir. Res..

[107] Corren J., Parnes J.R., Wang L., Mo M., Roseti S.L., Griffiths J.M., van der Merwe R. (2017). Tezepelumab in Adults with Uncontrolled Asthma. N. Engl. J. Med..

[108] Emson C., Corren J., Sałapa K., Hellqvist Å., Parnes J.R., Colice G. (2021). Efficacy of Tezepelumab in Patients with Severe, Uncontrolled Asthma with and without Nasal Polyposis: A Post Hoc Analysis of the Phase 2b PATHWAY Study. J. Asthma Allergy.

[109] Sverrild A., Hansen S., Hvidtfeldt M., Clausson C.M., Cozzolino O., Cerps S., Uller L., Backer V., Erjefält J., Porsbjerg C. (2021). The effect of tezepelumab on airway hyperresponsiveness to mannitol in asthma (UPSTREAM). Eur. Respir. J..

[110] Wechsler M.E., Menzies-Gow A., Brightling C.E., Kuna P., Korn S., Welte T., Griffiths J.M., Sałapa K., Hellqvist Å., Almqvist G. (2022). Evaluation of the oral corticosteroid-sparing effect of tezepelumab in adults with oral corticosteroid-dependent asthma (SOURCE): A randomised, placebo-controlled, phase 3 study. Lancet Respir. Med..

[111] Menzies-Gow A., Wechsler M.E., Brightling C.E., Korn S., Corren J., Israel E., Chupp G., Bednarczyk A., Ponnarambil S., Caveney S. (2023). Long-term safety and efficacy of tezepelumab in people with severe, uncontrolled asthma (DESTINATION): A randomised, placebo-controlled extension study. Lancet Respir. Med..

[112] Pitre T., Jassal T., Angjeli A., Jarabana V., Nannapaneni S., Umair A., Hussain M., Leung G., Kirsh S., Su J. (2023). A comparison of the effectiveness of biologic therapies for asthma: A systematic review and network meta-analysis. Ann. Allergy Asthma Immunol..

[113] Indolfi C., Dinardo G., Klain A., Contieri M., Umano G.R., Decimo A., Ciprandi G., Del Giudice M.M. (2023). Time effect of dupilumab to treat severe uncontrolled asthma in adolescents: A pilot study. Allergol. Immunopathol..

[114] Pelaia C., Busceti M.T., Crimi C., Carpagnano G.E., Lombardo N., Terracciano R., Vatrella A., Pelaia G. (2020). Real-Life effects of benralizumab on exacerbation number and lung hyperinflation in atopic patients with severe eosinophilic asthma. Biomed. Pharmacother..

[115] Pelaia C., Lombardo N., Busceti M.T., Piazzetta G., Crimi C., Calabrese C., Vatrella A., Pelaia G. (2021). Short-Term Evaluation of Dupilumab Effects in Patients with Severe Asthma and Nasal Polyposis. J. Asthma Allergy.

